# Cognitive Impairment in Heart Failure

**DOI:** 10.1155/2012/595821

**Published:** 2012-06-06

**Authors:** Efthimios Dardiotis, Gregory Giamouzis, Dimos Mastrogiannis, Christina Vogiatzi, John Skoularigis, Filippos Triposkiadis, Georgios M. Hadjigeorgiou

**Affiliations:** ^1^Department of Neurology, University of Thessaly, University Hospital of Larissa, P.O. Box 1400, Larissa, Greece; ^2^Department of Cardiology, University of Thessaly, University Hospital of Larissa, Larissa, Greece; ^3^TEI of Lamia, Lamia, Greece

## Abstract

Cognitive impairment (CI) is increasingly recognized as a common adverse consequence of heart failure (HF). Although the exact mechanisms remain unclear, microembolism, chronic or intermittent cerebral hypoperfusion, and/or impaired cerebral vessel reactivity that lead to cerebral hypoxia and ischemic brain damage seem to underlie the development of CI in HF. Cognitive decline in HF is characterized by deficits in one or more cognition domains, including attention, memory, executive function, and psychomotor speed. These deficits may affect patients' decision-making capacity and interfere with their ability to comply with treatment requirements, recognize and self-manage disease worsening symptoms. CI may have fluctuations in severity over time, improve with effective HF treatment or progress to dementia. CI is independently associated with disability, mortality, and decreased quality of life of HF patients. It is essential therefore for health professionals in their routine evaluations of HF patients to become familiar with assessment of cognitive performance using standardized screening instruments. Future studies should focus on elucidating the mechanisms that underlie CI in HF and establishing preventive strategies and treatment approaches.

## 1. Introduction

Heart failure (HF) is a major and growing health problem in the developed world that affects 1-2% of the adult population and 6–10% of people over the age of 65 [1, 2]. HF is associated with frequent hospital admissions, reduced quality of life, significant morbidity, and increased mortality [3–6]. It is estimated that elderly HF patients have high readmission rates ranging from 40 to 50% within 6 months [7]. Significant predictors of HF decompensation and high readmission rates include patients' poor compliance with therapy and diet restrictions, and their failure to recognize early symptoms of HF deterioration which may be the consequences of cognitive impairment (CI) and poor insight [8].

Several studies have demonstrated that CI is particularly common in HF with 30% to 80% of patients with HF experiencing some degree of cognitive impairment [9, 10]. This wide range in CI prevalence estimates is believed to be the result of diverse study designs, HF severity, age of patients, sample sizes, neuropsychological tests, and diagnostic criteria between different studies. HF adversely affects various aspects of cognitive functioning, including attention, learning ability and delay recall, working memory, executive function, and psychomotor speed [9–11]. Areas of cognition less affected are the language domain and possibly visuospatial functions although both domains have not been adequately investigated in patients with HF [12]. Most of the patients with HF and CI suffer from mild impairment in cognition whereas about 25% may have moderate-to-severe CI [9]. In addition, HF severity has been linked to increased risk of CI [13], while effective treatment of HF, use of ACE inhibitors, and physical activity lead to improvement in cognitive performance [14] which imply that CI may fluctuate in severity and can also be modified to some degree.

In this paper, we outline the spectrum of cognitive functional domains and describe the specific patterns of cognitive decline and their consequences in patients with HF. We also discuss the current understanding of the underlying mechanisms that affect neuronal function in HF and finally we provide suggestions for future research in this field.

## 2. Cognition and Cognitive Impairment

Cognition is a collective term for higher cortical functions such as thinking, remembering, knowing, planning, and analyzing. Cognition is crucial for a person to become aware of his/her situation, needs, and goals and meet the challenges of daily life [15]. Cognitive functioning encompasses various specific aspects referred to as cognitive domains that include memory, attention, executive functioning, psychomotor speed, language, and visuospatial ability. Positron emission tomography and functional MRI have shown that each cognitive domain involves diverse and often overlapping parts of the brain.

Several measures of cognitive functioning are available. A measure of global cognition that is often used by the clinicians as a screening instrument is the Mini-Mental State Examination (MMSE). MMSE is a 30-point test that provides information about orientation, working and episodic memory, attention, calculation, naming, copying, language comprehension, and visuospacial construction. Very often, however, a more detailed neuropsychological assessment is required. For this purpose, a number of neuropsychological tests are available, designed for the assessment of different cognitive domains and the calculation of cognitive dysfunction severity.

CI is a broad term that generally describes a decline in cognitive functions. The severity of this impairment may range from mild symptoms to severe cognitive deficits that may warrant the diagnosis of dementia. Mild cognitive impairment is described as a transition phase between normal ageing and dementia. This syndrome reflects the clinical situation in which a person has subjective complaints of CI as well as objective measurements of cognitive decline (around 1.5 standard deviations below normative data) along with intact daily functioning [16]. Mild cognitive impairment may involve single or multiple domain deficits (with or without memory impairment). Individuals with mild cognitive impairment are in increased risk of progression to dementia [17]. Dementia is characterized by progressive impairment in more than one cognitive domain. Routine laboratory tests for dementia include measurement of liver, renal and thyroid function, vitamin B12 levels, and imaging of the brain (CT or MRI). After excluding reversible causes, four common dementia syndromes, that is, Alzheimer's disease, vascular dementia, dementia with Lewy body, and frontotemporal dementia, account for 90% of all cases. These dementias have distinct clinical features, cognitive profiles, and imaging abnormalities [18].

## 3. Pathophysiology of CI in HF

The exact pathophysiologic mechanisms that underlie the development of CI in a proportion of patients with HF continue to be investigated and much research is conducted in the field. Studies have provided evidence that the clinically detected CI in patients with HF can be the outcome of structural or neurodegenerative changes which cannot be reversed and/or functional neuronal dysfunction which may progress to neuronal cell death or improve in response to treatment.

Cerebral and systemic hemodynamics seem to influence the development of CI in patients with HF. Cerebral blood flow (CBF), estimated with single-photon emission computed tomography (SPECT), was reduced about 30% in patients with severe HF (NYHA class III/IV) [19]. In another study the degree of CI in HF was related to regional CBF reductions particularly in the posterior cortical areas of the brain [20]. Interestingly, CBF in patients with severe HF was restored after heart transplantation [19]. These data suggest that cognitive performance in patients with HF appears to be closely related to the measurements of cerebral perfusion.

Cerebral perfusion is mediated by a number of factors including cardiac output and cerebrovascular reactivity. Low systolic blood pressure was shown to be an independent predictor of CI in HF patients [21]. In addition, low cardiac output was associated with impairment in cognitive performance [22–26] and dementia [27]. Furthermore, cerebrovascular reactivity, measured as the response to cerebral vasodilatory effects of carbon dioxide, was found to be impaired in patients with HF and correlated with left ventricular ejection fraction and NYHA class [28]. It appears therefore that low cardiac output, low systolic blood pressure, and impaired cerebral neurohormonal autoregulatory mechanisms in HF result in a decrease in cerebral blood flow that may account for the neuroanatomic and neuropsychological changes [29].

Another aspect of the pathophysiology of CI in HF is the development of cerebral abnormalities as a result of chronic hypoperfusion or stroke [30–33]. Cardiac output was shown to be associated with lower brain volumes and information speed processing [24]. In addition, some brain regions including the frontal cortex and parahippocampal gyrus, which are considerably implicated in cognition, seem to be more vulnerable in patients with HF [34]. MRI studies have also revealed [35] that HF patients have increased frequencies of focal brain abnormalities ranging from multiple cortical or subcortical infarcts to small vessel disease with white-matter lesions and lacunar infarcts with cerebral embolism and hypoperfusion being the most plausible mechanisms [36]. The location of the lesions in each individual with HF is likely to determine the domain specific impairment and these differences among patients with HF may account for the inconsistencies between the studies regarding the impairment of specific domains. In addition to location, lesion load and the ensuing cortical atrophy are also important determinants of cognitive impairment. Imaging techniques that integrate lesion location and burden would be valuable tools in predicting the cognitive consequences of HF.

## 4. Impairment of Cognitive Domains in Heart Failure

In most studies investigating the association between HF and cognitive performance using various neuropsychological tests, the term CI was used without specifying if the criteria for mild CI or specific dementias in each individual were met. In contrast, associations of CI, either global or in a specific domain, were searched by comparing the test scores between patients and controls or normative data [9, 11, 12, 37]. This approach may account for the inconsistencies between the studies regarding the prevalence of CI and the impaired domains. In the following section we briefly describe the main cognitive domains and the identified CI profiles in HF patients.

### 4.1. Memory

Memory is a basic cognitive function, which includes three main stages: registration, storage, and retrieval. Several types of memory exist, each involving different brain areas. The most important types for clinical use are the episodic memory which refers to the memory of specific personal events and experiences and the semantic memory which reflects the memory of meanings and general knowledge. Memory involves structures mainly within the medial temporal lobe, such as the hippocampal region, the entorhinal, perirhinal, and parahippocampal cortex [38]. Other regions related to memory include diencephalic nuclei, the mammillary bodies, portions of the thalamus, and prefrontal areas. Memory loss is a usual complaint of many elderly people. Interview questions regarding the patient's personal life and public events are useful for an initial screen of memory problems. The California Verbal Learning Test (CVLT) and the Rey Auditory Verbal Learning Test (AVLT) are memory tests that require learning and immediate recall (immediate memory) of lists of words repeatedly presented. Then a second list of words is presented. After some time has elapsed the patient is asked to recall the first list (delay memory) [39]. Episodic memory impairment, which is manifested as impaired ability to acquire, encode, and retrieve new information, is the main cognitive deficit in Alzheimer's disease and is directly related to mesial temporal lobe atrophy [40]. Semantic memory is assessed through category fluency tests, picture-naming tests and word-picture matching tests. Deficits in semantic memory may be present in Alzheimer's disease or more prominently in semantic dementia [41]. In HF several studies have revealed cognitive deficits in both the initial learning of information and the delay recall of that information at a later time point [11, 13, 42–53]. However, some other studies did not confirm a decline in initial learning scores [54–56].

### 4.2. Attention-Working Memory-Psychomotor Speed

 Attention is the ability to concentrate and focus selectively on a stimulus without being distracted by the background noise. Intact attention is essential for the patient's performance in other cognitive tasks. Working memory is a function of attention important for information processing. It refers to the ability to maintain and manipulate information temporarily in the mind and then retrieve it accurately in a few seconds, for instance, our ability to remember a phone number within a period of 30 seconds. The prefrontal cortex is considered the major brain structure involved in working memory. However, various other brain areas including parietal cortex, subcortical, and cerebellar regions also participate in working memory [57]. Serial subtractions of 7 from 100 are an easy and frequently used test for detecting attention problems. Attention and working memory can also be assessed using the digit span forwards and backwards and the Trail-Making-Test-A (TMT-A) in which individuals are instructed to connect sequential numbers by drawing lines. Impairment of attention and working memory is a feature of various medical conditions, including delirium and dementia. Another important aspect of cognition is the speed of information processing or else the psychomotor speed. It refers to the reaction time between a stimulus and the subsequent response. It represents a basic cognitive domain that affects other domains, especially executive functions. Processing speed is globally distributed in the brain and is dependent on the connectivity of nearly all cortical regions. It is believed that subcortical areas play an important role in this cognitive process [58]. Psychomotor slowing is characteristic of vascular, subcortical, and multi-infarct dementia [59, 60]. The Digit Symbol Substitution Test (DSST) is a useful test for the assessment of psychomotor speed. This test consists of 9 digit-symbol pairs. The patient is given an array of digits and is required to write the corresponding symbol beneath each digit as fast as possible. Another test used for the assessment of processing speed is the TMT-A and TMT-B. TMT-B also has a considerable executive function component. Deficits in attention, working memory, and speed of processing were detected in patients with HF in most studies [11–13, 42–47, 50, 52, 56, 61–64] but not all [44, 47, 48, 54, 65]. Given that these domains are mainly affected in vascular dementia it is possible that CI in HF and vascular dementia may share similar pathophysiologic mechanisms [66].

### 4.3. Executive Functions

 Executive functioning refers to the cognitive abilities needed for daily life. Under this term, a wide range of cognitive processes and behavioral competencies are included, some of them being verbal reasoning, problem solving, planning, multitasking, cognitive flexibility and the ability to deal with novelty [67]. These skills primarily involve the frontal and prefrontal cortex. Complex cortical and subcortical circuits were also recognized to participate in executive functioning [68]. Deficits in executive functions are associated with a decline in one's ability to fulfill the requirements of everyday life. These deficits are common and occur early in the course of frontotemporal and vascular dementia. A number of tests are available for evaluating executive functions. The Stroop Test, the TMT-B, the letter fluency test and the Wisconsin Card Sorting Test (WCST) are commonly, used tests in clinical studies [69]. In patients with HF, the majority of studies found significant impairment in measures of executive functioning [11, 43, 44, 49, 51, 52, 61, 64], whereas two studies did not confirm such deficits [13, 20].

### 4.4. Language

 Language deficits are quite common in dementias. Patients usually complain of difficulties in word finding and naming. They may use related words instead of the target word, have trouble following instructions or staying on a conversation. Patients also have impairment in word recognition in reading and writing [70]. The main brain regions for language are Broca's area, which is related to motor aspects of speech and Wernicke's area, which is responsible for language perception. These two regions are connected to each other and to temporal, prefrontal, and parietal regions, forming a complex network involved in language function. Evaluation of language domain includes assessment of naming, repetition, following commands, verbal fluency, reading and writing. Boston Naming Test (BNT), Benton Controlled Oral Word Association Test (COWAT), Token Test, and semantic fluency task are some useful neuropsychological tests for examination of language function. It should be noted that language disturbance can interfere with the patient's performance in other cognitive domains. Compared to other domains, there has been little work on language deficits in patients with HF. Two studies reported impaired performance on language measures in patients with HF [20, 52].

### 4.5. Visuospatial Function

 Visuospatial function refers to the visual perception of the environment and the spatial relationships between the objects. Common manifestations of visuospatial deficits include impairment in navigation and topographical orientation and difficulty in dressing, recognizing familiar faces, or grasping objects [71]. The neural network that mediates visuospatial cognition is widely distributed and includes areas of parietal lobes, occipital cortex, lateral prefrontal cortex, medial and inferior temporal cortex, basal ganglia, and white-matter tracts. Neuropsychological tests for the assessment of visuospatial functions include Benton Facial Recognition Test (FRT), Judgement of Line Orientation Test (JLO), and Block Design Test. The Clock Drawing Test (CDT) is also widely used for evaluation of visuospatial and constructional ability. Deficits in visuospatial perception and construction are a prominent manifestation of Alzheimer's disease. In patients with HF some studies noticed visuoperceptual deficits [47, 49, 53]. Another study, however, did not replicate this finding [52].

Given the high occurrence of deficits in cognitive function in patients with HF and their prognostic value in the course of HF, adequate assessment and early detection of CI is essential. HF patients with CI may have diverse patterns of cognitive domain involvement which can be understood considering the pathophysiology of CI in HF where a concomitant and uneven involvement of multiple brain areas occurs. All these cognitive domains should be included in the assessment of CI in HF in order to achieve both reliability and sensitivity.

Most studies have used various measures of cognitive functioning, and currently there is no consensus among investigators regarding the optimum neuropsychological tests to assess patients with HF. Brief screening instruments such as the MMSE or the Montreal Cognitive Assessment (MoCA) can be easily administered by health professionals in the outpatient clinical practice to confirm the presence of cognitive impairment. Brief tests, however, may be insufficient in identifying subtle cognitive deficits and more detailed neuropsychological assessment will be required. On the other hand, implementation of comprehensive series of tests requires specific training for administration and interpretation, is time consuming, and consequently may affect performance and compliance of participants. Future studies therefore should focus on validating a screening instrument that combines brevity, ease of use, and sensitivity to detect in HF patients the presence of cognitive impairment in each specific domain.

## 5. Severity Progression of CI

The prevalence of CI severity in HF patients with CI has not been adequately addressed in the literature. In addition, no standard criteria were used for delineating between mild, moderate, and severe CI in the studies. Most of the patients with HF and CI in these studies had mild impairment in cognition, whereas about 25% had moderate-to-severe CI [9, 56, 72]. Furthermore, the degree of cognitive decline in HF patients appears to correlate with the severity of HF. Zuccalà et al. [23] in a study of 57 patients with HF described a nonlinear relationship between left ventricular ejection fraction and MMSE scores. Interestingly, a greater decrease in rate of MMSE scores was revealed for ejection fraction (EF) values <30%. A similar relationship between measures of verbal memory and EF drop below 30% was noted by another group in patients older than 63 years [73]. Likewise, other studies found that decrease in cardiac output [21, 25], long duration of HF [74], and higher New York Heart Association (NYHA) class of the disease [75] parallel the severity of cognitive impairment.

Another important issue which has received much attention lately is whether cognitive impairment in patients with HF progresses to dementia over time, remains stable, or even remit. In an important population-based cohort study [27] Qiu et al. investigated the progression to dementia of 205 individuals with HF over a 9-year follow-up period. At baseline assessment HF patients, although nondemented, had lower cognitive performance measured by MMSE test compared to controls. These patients, in the long-term follow-up, were at increased risk of progression to dementia (HR: 1.84, C.I.: 1.35–2.51) or Alzheimer's disease (HR: 1.80, C.I.: 1.25–2.61). Interestingly, the use of antihypertensive drugs seemed to counteract the effect of HF on dementia risk. Another study in patients aged >80 years examined the relationship between HF and the change over time of specific cognitive domains. It was shown that measures of episodic memory decline more in HF patients compared to the group without HF [47].

Finally, some studies reported improvements in cognitive performance of HF patients over time which could be attributed to improvement of cerebral hypoperfusion and consequently neuronal function due to optimum management of heart failure [76, 77], initiation of ACE inhibitors [14], or implementation of exercise training programs [64].

## 6. Impact of CI in HF

Deficits in attention, learning, memory, executive functions, and psychomotor speed observed in high prevalence in patients with HF may impair their ability to carry out self-care and adhere to treatment requirements. Such deficits may also compromise patients' capacity to recognize HF worsening symptoms and make appropriate decisions about their health care [8]. In a recent study [78] mild CI was a significant predictor of lower self-care management and self-confidence scores with other determinants of self-care being severity of HF and presence of comorbidities. In addition, HF patients with CI had greater difficulty with medication management [79], were less likely to participate in outpatient treatment programs [74], and failed to recognize early symptoms and make the appropriate self-care decisions [80, 81]. As a result, these patients were at increased risk of HF decompensation, unplanned hospital admissions or even death [82]. These data suggest that CI may ultimately represent a risk factor of suboptimal health care and worse outcome of HF patients. Impairments in attention, judgment, and speed of information processing should be taken into account when treatment strategies are planned.

A number of studies in patients with HF also examined the impact of CI on disability in daily activities, mortality, and quality of life. The functional independence in daily activities was investigated in a large multicentre study in patients with HF. The study revealed that CI was associated with a sixfold increase in functional disability (OR: 6.49; 95% C.I.: 4.39–9.59) independently of any potential confounders such as age, sex, comorbidities, medications or low blood pressure [83]. Dependence and increased disability are known predictors of raised mortality and increased hospital readmission rates. CI was also associated with increased risk of mortality. In a study investigating the in-hospital mortality among HF patients, CI was found to increase the mortality by five times (Relative Risk (RR): 4.9; 95% C.I.: 2.9–8.3) after adjusting for multiple confounders [84]. CI, therefore, seems to represent a hidden comorbidity with an adverse impact on disease course, influencing the burden of disease, survival rates, and resource consumption. Finally, cognitive decline that accompanies HF may negatively affect many aspects of daily life and the patients' perceptions of quality of life and well-being. In a recent study [85], significant determinants of the patients' quality of life included disease severity, age, depressive symptoms, and memory measures although the latter accounted only for a small amount of the observed variance.

## 7. Therapeutic Implications

A small number of studies have addressed the influence of HF treatment on cognitive performance of patients with HF. In a retrospective database analysis of 1220 patients with HF, the use of ACE inhibitors was associated with improvement in cognitive performance (OR: 1.57; 95% C.I.: 1.18–2.08). Furthermore, the probability of improvement increased with higher dosages of ACE inhibitors and longer duration of treatment [14]. In addition, the same group of investigators reported [86] that the use of digoxin also improved cognitive performance among older patients with HF, reaching an OR of 1.69 (95% CI: 1.20–2.38). In another study of 50 patients with severe HF that were reassessed 6 weeks after the introduction of the appropriate medications, it was shown that effective treatment of HF patients according to their needs with diuretics, ACE inhibitors, cardiotonic medication (such as digoxin), and antiarrhythmic drugs had beneficial effects in patients' cognitive performance and in particular in attentional and visuospatial scores [87]. These studies provided valuable evidence that specific medications used in HF such as ACE inhibitors and digoxin as well as optimal HF therapy may have beneficial effects on cognitive performance of HF patients. However, further research is needed mainly from randomized trials in order to confirm these results and establish possible favorable role of various medications on cognition.

Some studies also examined the role of nonpharmacological approaches on cognition in patients with HF. In agreement with pharmacological treatments invasive methods were also demonstrated to improve cognition. It was reported that impaired cognitive function in patients with HF was significantly improved after heart transplantation [42, 65]. Of note, pacemaker implantation in bradycardic patients was associated with improvement in verbal cognitive function [88]. Two studies tested the impact physical activity on cognitive function of HF patients. As expected, physical activity was demonstrated to have beneficial effects on cognitive performance [64, 89]. Finally, the efficacy of cognitive training intervention known as cognitive rehabilitation on mental performance was evaluated in patients with HF. The authors through structured cognitive training programs designed to improve several aspects of cognition found significant improvements on measures of working memory, psychomotor speed, executive function, and memory [90]. These interesting results should be further investigated especially in larger randomized controlled trials.

There are no current studies examining the effects of the acetylcholinesterase inhibitors (i.e., the group of medications licensed for the symptomatic treatment of Alzheimer's disease) on cognitive performance of patients with HF. Given that acetylcholinesterase inhibitors were also found efficacious in vascular dementia, a similar effect might be expected in patients with HF. Other approaches such as repetitive transcranial magnetic stimulation [91] could also be considered for CI in HF.

In summary, optimal HF treatment through pharmacological or invasive approaches ameliorates cognition among patients with HF. Effective control of vascular risk factors should also be achieved considering the pathophysiology of CI in HF. Patients should be encouraged to participate in cognitive, physical, and social activities in order to improve their cognitive performance.

## 8. Conclusions

Cognitive impairment is particularly common in HF and is increasingly regarded as an independent prognostic factor of HF outcome since it exerts significant effects on quality of life, disability, morbidity, and mortality of patients with HF.

HF patients may present with deficits in attention, learning ability and delay recall, working memory, executive function, and psychomotor speed. They may have fluctuations in their cognition over time, depending on the effective management of HF, but they are at increased risk of progression to dementia. However, more conclusive evidence especially from prospective longitudinal studies is required for the establishment of the long-term course of CI in HF patients and the possibility of emergence of modifiable risk factors and new targets for intervention.

CI in HF seems to result from structural changes in the brain cortex and white-matter due to microembolism, chronic or intermittent cerebral hypoperfusion, and impaired flow regulation in small vessels which lead to cerebral hypoxia and ischemic brain damage. Along with the neurodegenerative component, there is also a functional and consequently modifiable component of neuronal dysfunction due to decreased cerebral blood flow which may account for the improvements of cognitive performance after effective treatment of HF. Future studies are needed to provide further insights into the relationship between CI and tissue structural abnormalities and neuronal dysfunction.

Furthermore, a priority for researchers is the identification of a screening instrument sensitive to the cognitive profile of HF and easy to use in the clinical setting. With increasing awareness that CI can worsen HF outcome, health professionals should recognize the importance of early identification and management of patients at risk of CI and become familiar with assessment of cognitive performance in their routine evaluations.
